# A Descriptive Analysis of Urinary ESBL-Producing-*Escherichia coli* in Cerdanya Hospital

**DOI:** 10.3390/microorganisms10030488

**Published:** 2022-02-22

**Authors:** Lorena Patrícia Gaviria, Lourdes Montsant, Carlos Azuaje, Aida González-Díaz, Juan P. Horcajada, Enric Limón, Miguel Viñas, Paula Espinal, Ester Fusté

**Affiliations:** 1 Internal Medicine Service, Hospital Transfronterer de Cerdanya, Puigcerdà, 17520 Girona, Spain; loregama89@gmail.com (L.P.G.); lmontsant06@gmail.com (L.M.); azuaje@sapo.pt (C.A.); 2 Microbiology Department, Hospital Universitari de Bellvitge, IDIBELL, Hospitalet de Llobregat, 08907 Barcelona, Spain; agonzalezd@bellvitgehospital.cat; 3 Ciber de Enfermedades Respiratorias (CIBERes), ISCIII, 28020 Madrid, Spain; 4 Department of Infectious Diseases, Hospital del Mar, Institut Hospital del Mar d’Investigacions Mèdiques (IMIM), 08003 Barcelona, Spain; jhorcajada@psmar.cat; 5 Programa VINCat, Institut Català d’Oncologia, Hospitalet de Llobregat, 08907 Barcelona, Spain; elimon@iconcologia.net; 6 Department of Public Health, Mental Health and Perinatal Nursing, US of Nursing, University of Barcelona, Hospitalet de Llobregat, 08907 Barcelona, Spain; 7 Laboratory of Molecular Microbiology & Antimicrobials, Department of Pathology & Experimental Therapeutics, Bellvitge Institute for Biomedical Research (IDIBELL), Medical School, University of Barcelona, Hospitalet de Llobregat, 08907 Barcelona, Spain; mvinyas@ub.edu

**Keywords:** *Escherichia coli*, multidrug resistance (MDR), extended-spectrum β-lactamase (ESBL), urinary tract infections (UTI), molecular characterization, whole-genome sequencing (WGS), sequence type (ST)

## Abstract

Urinary tract infections caused by extended-spectrum β-lactamase *Escherichia coli* (ESBL-EC) are increasing worldwide and are a current concern because treatment options are often limited. This study investigated antimicrobial susceptibility, antimicrobial resistance genes (ARGs), and the biological diversity of urinary ESBL-EC isolates at Cerdanya Hospital, a European cross-border hospital that combines French and Spanish healthcare models. Bacterial identification and susceptibility were determined using the Microscan WalkAway^®^ system and ESBL production was examined by the double-disk synergy method. Isolates were sequenced using the Ion S5^™^ next-generation sequencing system, with the whole-genome sequences then assembled using SPADEs software and analyzed using PubMLST, ResFinder, FimTyper, PlasmidFinder, and VirulenceFinder. A phylogenetic analysis was performed by constructing an assembly-based core-SNV alignment, followed by a phylogenetic tree constructed using Parsnp from the Harvest suite. All isolates studied were multidrug-resistant and could be classified into 19 different sequence types characterized by a high genetic diversity. The most prevalent ESBL-enzymes were CTX-M-14 and CTX-M-15. High-risk international clones (ST131, ST10, and ST405) were also identified. The results demonstrated the absence of a single predominant clone of ESBL-MDR-EC at Cerdanya Hospital.

## 1. Introduction

Urinary tract infections (UTIs) are among the most common human infections and can be both nosocomial and community-acquired. These infections affect individuals of all age groups and are, particularly in elderly, a cause of morbidity in out-patients as well as hospitalized patients. The so-called uropathogenic *Escherichia coli* (UPEC) strains are the primary etiological agents; more than 90% of these infections are community-acquired, with the rest being hospital-acquired

UTIs caused by multidrug-resistant *Escherichia coli* able to produce extended-spectrum β-lactamases (ESBL-MDR-EC) are increasing worldwide. β-lactamases naturally occur in some bacterial species; nevertheless, they may be mobilized by plasmids and have become widespread, in part as a consequence of the use, abuse, and misuse of β-lactam antibiotics. In Gram-negative bacteria, TEM-1 and SHV-1, two broad-spectrum β-lactamases, have greatly increased in frequency as a result of the introduction of first- and second-generation cephalosporins. This situation was the driver of the development and introduction of new classes of β-lactams resistant to hydrolysis by these enzymes (the so called expanded-spectrum β-lactam antibiotics). These drugs are nowadays frequently used. As expected, this has driven a new evolutionary step with the emergence of new enzymes able to hydrolyze the new β-lactams. ESBL genes are present in many genera of enterobacteria and in *Pseudomonas aeruginosa*, and encode for enzymes hydrolyzing expanded-spectrum cephalosporins (such as ceftazidime or cefotaxime) and monobactams (aztreonam). Originally, ESBLs were variants of TEM and SHV, although multiple variants have been identified [1].

Due to the scarcity of available therapeutic options, these infections constitute a challenge for public health in both the community and the hospital setting [2,3] as they may lead to significant mortality and morbidity, especially in immunocompromised and very elderly patients [4]. The significant economic impact of ESBL-MDR-EC infections has been attributed to high antibiotic costs and the high-level use of healthcare resources [2,5,6].

A broad diversity of *E. coli* isolates that cause UTIs has been demonstrated, including internationally identified high-risk clones such as *E. coli* sequence type (ST) 131 [7,8], as well as ST69, ST10, ST405, and ST38 [9]. The success of most of these STs is due to features relevant for their pathogenesis, including the ability to evade host immunity, multi-drug resistance, and the presence of several virulence factors (VFs); certain O:H serotypes known to be particularly virulent [3,10,11].

The resistance of *E. coli* to third-generation cephalosporins (3GC) has increased over the last decade. In Spain, *E. coli* resistant to 3GC increased from 12.1% in 2010 to 14.1% in 2020, according to the ECDC (http://atlas.ecdc.europa.eu/public/index.aspx, accessed on 22 December 2021). In addition, the incidence of UTIs caused by ESBL-producers is at least 40% [8]. In Spain, the increase of these UTIs in non-hospitalized individuals has been driven by the overuse and the misuse of antibiotics in human and veterinary medicine, as well as in agriculture [12,13,14].

The genomic plasticity of *E. coli*, including its ability to acquire both resistance and virulence genes, has been investigated by approaches that include whole-genome sequencing analysis (WGSA). As we lack fossil record of prokaryotes and evolution may in some circumstances be very fast, several methods have been proposed to estimate changes in diversification rates through time and across lineages from phylogenetic data [15].

In this study, WGSA was used to characterize ESBL-producing *E. coli* isolates recovered from a European cross-border hospital between 2016 and 2017. The molecular epidemiology, antimicrobial resistance genes (ARGs), plasmids, and VFs of the identified STs were determined as well.

## 2. Materials and Methods

### 2.1. Patients and Bacterial Isolates

This study was conducted at Hospital de Cerdanya, the first cross-border hospital in Europe. The hospital covers an area of more than 1300 km^2^ and is responsible for the care of 33,000 inhabitants (permanent population), spread among 53 municipalities located in the highlands on either side of the border. As the region is also popular with tourists, the winter and summer populations are often at least four-fold higher. Annual admissions to Cerdanya Hospital number are around 27,000.

A total of 533 isolates of *E. coli* were obtained from patients (>18 years) admitted to the hospital with a UTI between 2016 and 2017. Almost all studied infections (*n* = 24) were classified as community acquired; only two were hospital-acquired. A total of 33 isolates of *E. coli* had reduced susceptibility to second-generation cephalosporins (i.e., cefoxitin, cefuroxime) and 3GC (i.e., cefotaxime and ceftazidime), and were screened for ESBLs. Those isolates recovered from the same patients were included in the study, given the different antimicrobial susceptibility profiles of the isolates (Table S1).

### 2.2. Bacterial Culture, Antimicrobial Susceptibility Testing, and ESBL Detection

Isolates were recovered by conventional microbiological methods. *E. coli* isolates were identified using MicroScan (MicroScan WalkAway^®^, Beckman Coulter, Inc., Brea, CA, USA). Antibiotic susceptibility testing was performed by broth microdilution and interpreted according to the European Committee for Antimicrobial Susceptibility Testing (EUCAST) clinical breakpoints [16]. The isolates were considered as MDR following the criteria defined by Magiorakos et al. [17]. ESBL production was confirmed using the modified double-disk synergy test [18]. Among the 33 presumptively ESBL-producer isolates, seven did not display ESBL genes after WGSA and subsequently were removed from the study.

### 2.3. Whole-Genome Sequencing and Bioinformatic Analysis

Genomic DNA of the confirmed ESBL-EC isolates was extracted using the DNeasy tissue kit following the manufacturer’s instructions (Qiagen, Hilden, Germany) and sent to CosmosID (Rockville, MD, USA) for WGS using the Ion S5™ next-generation sequencing system (Thermo Fischer Scientific, Waltham, MA, USA). Genomes were assembled with SPAdes [19] and genome quality was assessed using Check-M-point [20].

Multilocus-sequence-typing was carried out using the *E. coli* Achtman scheme of PubMLST (https://pubmlst.org/, accessed on 20 December 2021). STs were determined using Enterobase (https://enterobase.warwick.ac.uk, accessed on 20 December 2021). Assembled genomes were analyzed using ResFinder v.4.1 [21,22,23], FimTyper v.1.0, PlasmidFinder v.2.1 [24], and VirulenceFinder v.2.0 [25], available from the Centre for Genomic Epidemiology (http://www.genomicepidemiology.org/, accessed on 20 December 2021). *E. coli* success in urinary tract colonization strongly depends upon the positivity for the following markers: *chuA*, *fyuA*, *vat*, and *ycfV* [26]. Serotype, the presence of the *fimH* allele, and phylogenetic groups were identified using SerotypeFinder 2 [25], FimTyper [27], and Enterobase [28], respectively.

The phylogenetic analysis was performed by constructing an assembly-based core-SNV alignment using Parsnp from the Harvest suite [29] with default parameters, with the exception of parameter -c. Isolate 8778 was selected as the reference genome and the total number of SNPs was obtained using HarvestTools. The WGS tree was visualized using iTOL 5 (https://itol.embl.de/upload.cgi, accessed on 28 January 2022) [30].

### 2.4. Sequence Data Deposition

All raw reads generated were deposited in the National Centre for Biotechnology Information (NCBI) under the BioProject number PRJNA746954.

## 3. Results

### 3.1. Bacterial Isolates and Antimicrobial Susceptibility

*E. coli* isolates in this study (*n =* 26) were obtained from 30 patients seen at the Hospital de Cerdanya. From two patients, two and three isolates, respectively, were included. Twenty-one isolates were obtained from women who presented community-acquired infections and cystitis as the most frequent diagnosis. Primary care and internal medicine were the most common hospital services. The remained isolates were from men treated at the emergency services, mainly with community acquired infection and diagnosed with acute bacterial prostatitis (https://uroweb.org/guideline/urological-infections, accessed on 28 January 2022) (Table S1).

Antimicrobial susceptibility testing identified all *E. coli* isolates as MDR. In addition to cefotaxime resistance, 14 of the isolates were resistant to ceftazidime, 21 to ciprofloxacin (cipR), and 16 to trimethoprim/sulfamethoxazole. Six isolates (30.3%) were resistant to amoxicillin/clavulanic acid, 12 to tobramycin, and nine to gentamycin. All isolates were susceptible to imipenem and to the combination of the β-lactam antibiotics piperacillin and tazobactam.

An analysis of the ARG content of the isolates showed that the β-lactam resistance gene *bla*_CTX-M_ was the most prevalent, found in 21 isolates, followed by *bla*_OXA-1_ and *bla*_SHV-12_ in six and five isolates, respectively. From the CTX-M family, *bla*_CTX-M-14_ (*n =* 9), *bla*_CTX-M-15_ (*n =* 6), and *bla*_CTX-M-3_ (*n =* 2) were the most frequent; concerning the TEM family, *bla*_TEM-1B_ (*n =* 12) and *bla*_TEM-1C_ (*n =* 2) were the most common.

CipR was associated with mutations in the quinolone-resistance-determining region (QRDR) in the chromosomal *gyrA* and *parC* genes. In *gyrA*, S83L was the predominant mutation (*n =* 22). A second mutation (D87N) was detected in 19 of these isolates and only one carried the mutations S83L and D87Y. In *parC*, S80I was the most common mutation (*n =* 15), with three of these isolates also carrying a second mutation: three E84V, one E84G, one E62K, and one A56T. Among the isolates with mutations in *parE*, S458A was identified in eight, I529L in three, and S458T in two. The triple mutation *gyrA* (S83L and D87N or D87Y) and *parC* (S80I) was found in 15 isolates. CipR was also related to the presence of *aac(6’)-Ib-cr,* found in seven isolates. All isolates with *bla*_CTX-M-15_ simultaneously contained the ARGs *bla*_OXA-1_ and *aac(6’)-Ib-cr*.

Trimethoprim/sulfamethoxazole resistance was mainly associated with the combinations of *drfA* (*n =* 18) and *sul* (*n =* 19) genes. *Sul1* alone was present in six isolates, *sul1* plus *sul2* in five, and *sul1* plus *sul3* in three.

In addition, *dfrA17* was the most frequent (*n =* 10), followed by *dfrA*36, *dfrA*16, *dfrA*14, and *dfrA*5, which occurred with less frequency.

Among the aminoglycosides, resistance was mainly related to the presence of the *aadA5* and *aph(6)-Id* (*n =* 9, each), *aadA2b* (*n =* 7), and *aac(6′)-Ib-cr* (*n =* 7) genes. Details of the antimicrobial susceptibility tests and AGR content of all the isolates are provided in Table S1.

### 3.2. Plasmid Typing

PlasmidFinder analysis confirmed the presence of a large diversity of plasmid replicons in the studied isolates. Among the 18 different replicons identified, the most common were IncFIB (*n =* 23), IncFII (*n =* 17), IncFIA (*n =* 10), IncI1 *(n =* 9), IncX1 (*n =* 6), IncFIC, and Col (*n = 5*, each). Most of the isolates contained more than one replicon; 13 harbored three, while seven had four, and two more than four replicons. IncN was detected once, but IncM and IncA/C were absent. Only one isolate was negative for replicons, although it was nonetheless resistant to some antimicrobial agents.

It should be noted that all isolates producing CTX-M-15 presented FIA, FIB, and FII, and some of them presented IncX1, while those producing CTX-M-14 presented a variation in the content of IncI1, IncX1 and IncF replicons, with FIA as the less prevalent (Table S1).

### 3.3. Virulence Factors

VirulenceFinder and FimTyper analysis revealed the presence of several VFs (*n =* 56) among the isolates: 0–10 VFs (*n =* 7), 11-20 VFs (*n =* 11), and 21–32 VFs (*n =* 8). The most frequent virulence genes encoding adhesins were *fim, pap, afa*, and *iha.* Genes encoding toxins were *hly, cnf, sat*, and *vat*, and genes related with iron uptake were *sitA, iucc, irp, chuA*, and *iroN*. Four of the isolates may be regarded as genuine uropathogenic *E. coli* (UPEC) carrying *chuA*, *fyuA, yfcV*, and/or *vat* (Table S1).

### 3.4. Molecular Epidemiology of the Strains

According to the MLST analysis, 19 different STs were found. The most prevalent were ST131 and ST44 (*n =* 3, each), followed by ST10, ST88, and ST405 (*n =* 2, each). The other STs comprise only one isolate each. ESBL-MDR-EC isolates were assigned mainly to the phylogenetic group A (*n =* 9), followed by B2 (*n =* 6), and B1 (*n =* 2). The most common replicons were from the IncF family and they were present in all isolates belonging to high-risk clones (ST131, ST405, and ST10) (Table 1 and Table S1).

**Figure 1:**
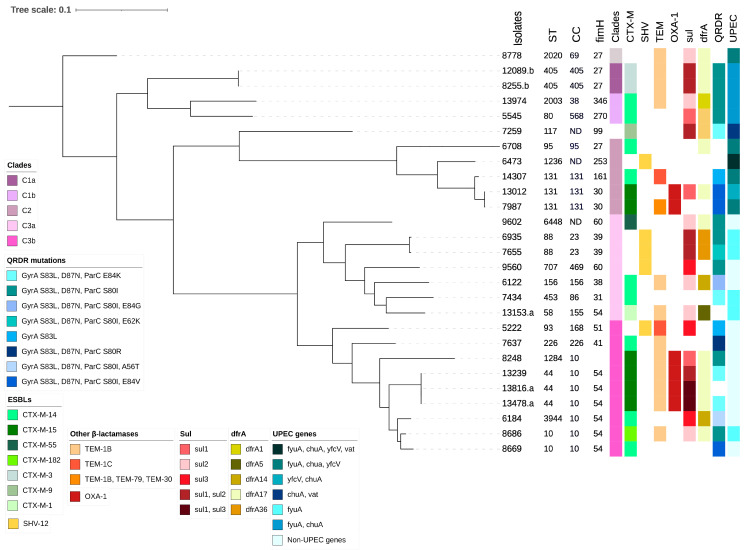
Phylogenomic analysis of *E. coli* isolates. Maximum likelihood tree of the 26 ESBL-MDR-EC isolates recovered from patients admitted to the Hospital de Cerdanya with a UTI during 2016 and 2017. The tree was rooted with *E. coli* 8778 ST2020. The clonal complex (CC), sequence type (ST), clade, antimicrobial resistance markers, and UPEC genes (*vat*, *fyuA*, *chuA*, and *yfcV*) relevant to the efficient colonization of the urinary tract are shown to the right of the tree.

A high clonal diversity was determined among the isolates, which were organized in three phylogenetic groups (C1, C2, and C3) and four subgroups (C1a, C1b, C3a, and C3b) (Figure 1). A core genome (in 60%) consisting of 5,333,101 bp, including 189,993 SNPs, was determined by mapping and alignment of the isolates to the reference strain *E. coli* 8778 (Figure 1).

The phylogenetic group C1 included four isolates recovered between April 2016 and November 2017 and was found to have caused community-acquired UTIs. In C1, with its subgroups C1a and C1b (Figure 1), between 18 and 35,974 SNPs were detected. The C1a phylogroup included two isolates (one susceptible to tobramycin and the other resistant) recovered from the same patient. Both isolates belonged to the high-risk clone ST405, serotype O102:H6, an unknown phylogroup. Besides the *fimH27* allele, they carried *bla*_CTX-M-3_ and *bla*_TEM-1B_, and were trimethoprim/sulfamethoxazole-resistant, which was conferred by both *sul1* and *sul2*. C1a isolates were also CipR and harbored mutations in *gyrA* (S83L and D87N) and *parC* (S80I). The replicon-types identified in this ST belonged to the IncF family (Table 1). Phylogroup C1b included two isolates, one belonging to ST2003 and the other to ST80. Both isolates carried *bla*_CTX-M-14_ and were CipR, associated with mutations in *parC* (S80I) and *gyrA* (S83L), with the latter being either D87N or D87Y.

ESBL-MDR-EC phylogroup C2 included six isolates, recovered between June 2016 and December 2017, that caused both community (*n =* 4) and healthcare-associated (*n =* 2) UTIs. Isolates within this clade showed high diversity (SNP range 88–70,988). The high-risk clone ST131 was identified in three isolates, and the highly pathogenic *E. coli* ST95 in one. This isolate belonged to phylogroup B2 and serotype O50:H4, and carried *fimH27* and *bla*_CTX-M-14_. Moreover, it was trimethoprim/sulfamethoxazole- and ciprofloxacin-susceptible. The other isolates were ST1236 and ST117.

Isolates from ST131 belonged to the lineage B2, O25:H4 serotype; among them, two had the *fimH30* allele, carried *bla*_CTX-M-15_ with *bla*_OXA-1_, and were CipR associated with the *aac(6’)-Ib-cr* gene and with mutations in *parC* (S80I and E84V), *gyrA* (S83L and D87N), and *parE* (I529L). In addition, they were related with hospital-acquired infections. The other ST131 isolate carried the *fimH161* allele and *bla*_CTX-M-14_ and *bla*_TEM-1C_. Only one ST131 isolate was trimethoprim/sulfamethoxazole-resistant, via *sul1* and *dfrA17*; CipR was associated with mutations in *gyrA* (S83L) and *parE* (S458A and I529L). EC 7987, isolated from a French patient in October 2016, showed a low genetic diversity (88 SNP) and was similar to EC 13012, isolated from a Spanish patient in September 2017. Both isolates were from patients with healthcare-associated UTIs. However, despite the similarity of their ARGs and VFs, it was not feasible to determine the mode of transmission, due to the lack of detailed epidemiological data.

ESBL-MDR-EC phylogroup C3 included 16 isolates obtained from March 2016 to November 2017, all of which caused community-acquired UTIs. C3 was divided into subgroups C3a (*n =* 7) and C3b (*n =* 9) (Figure 1). The six STs identified in subgroup C3a, ST6448, ST88, ST707, ST453, ST58, and ST156, contained between 671 and 72,250 SNPs.

There was no predominant phylogroup, serotype, or *fimH* allele among these isolates, and they encoded several *bla* genes, including *bla*_TEM-1B_*, bla*_SHV-12_, *bla*_CTX-M-1,_ *bla*_CTX-M-1_, and *bla*_CTX-M-55_. CipR (*n =* 6) was related to a double mutation in *gyrA* (S83L and D87N) and variations in *parC* (S80I, E84K and E84G) and *parE* (S458A) (Table S1).

The C3b phylogroup included isolates of the high-risk clonal complex (CC) 10, with different STs: ST10 (*n =* 2), ST44 (*n =* 3), ST1284, and ST3944. They presented a high genetic diversity (6–60,629 SNPs), with ST44 isolates containing between 6 and 10,490 SNPs and isolates within ST10 containing 3675 SNPs. The most predominant serotype was O101:H4, identified in all ST44 isolates.

All CC10 isolates belonged to phylogroup A, and six carried the *fimH54* allele. Four carried *bla*_CTX-M-15_ with *bla*_OXA-1_ and *bla*_TEM-1B_, and two isolates carried *bla*_CTX-M-14._

All C3b isolates were CipR with mutations in *gyrA* (S83L and D87N), *parC* (S80I, E84V, E84K, S80R and A56T)), and *parE* (S458A and S458T). In addition, CTX-M-15-producers also had the *aac(6′)-Ib-cr* gene (Table S1).

Overall, phylogroup C3b contained IncF replicons (FIB, FIA and FII), but also three and four isolates contained IncI1 and IncX1, respectively.

Virulence gene content was variable among all phylogroups; however, isolates of ST88 and ST131 had a high number [22,23,24,25,26,27,28,29,30,31,32] of genes, whereas isolates belonging to ST453, ST10, ST44, ST1284, ST6448, and ST226 had fewer VFs [2,3,4,5,6,7,8,9,10,11,12,13,14,15,16].

## 4. Discussion

UTIs represent a clinical problem in both community and hospital settings [7]. Community-acquired UTIs are the most common infection caused by ESBL-producing *E. coli* and have been increasing worldwide [31], which is consistent with the findings of this study, where 92% of the isolates were related to community-acquired infections. This phenomenon might represent a high risk of transmission to hospitalized patients who can become infected or colonized by these isolates [31]. The prevalence of ESBL-MDR-EC in UTIs and the difficulties in treating these patients constitute a challenge in infectious disease. In addition, the published studies differ greatly in their patient populations and antibiotic policies. There is also a need for studies that consider both community and hospital settings.

The prevalence of ESBL-MDR-EC in UTIs diagnosed at Hospital de Cerdanya between 2016 and 2017 (around 6%) was similar to that reported by other Spanish and French hospitals [10,13].

Moreover, ESBL-EC with an MDR profile is increasing at both the hospitalized and community level [7]. Accordingly, all isolates of this work were classified as MDR, which is difficult to treat in patients and constitutes a challenge in infectious disease management.

Overall, the antimicrobial resistance of the studied isolates was observed to be high to second- and third-generation cephalosporins (mainly cefotaxime and ceftazidime), ciprofloxacin and trimethoprim/sulfamethoxazole, similar to the results reported by other authors [31,32].

Among ESBL-producers, resistance to cefotaxime was found in more isolates than resistance to ceftazidime (100% and 53.8%, respectively), in accordance with the results reported by Oteo et al., who also found more resistance to cefotaxime than to ceftazidime (66.7% and 33.6%, respectively) [33].

ARGs content obtained by WGSA showed strong agreement with the phenotype-based resistance to all tested antimicrobial agents.

CTX-M-15 and CTX-M-14 β-lactamases are the most frequently occurring ESBLs worldwide [1]. In our work, CTX-M enzymes were the most prevalent among the studied isolates. While *bla*_CTX-M-15_ is considered to be predominant, followed by *bla*_CTX-M-14_, among our isolates, *bla*_CTX-M-14_ was more frequent than *bla*_CTX-M-15_, in agreement with previously reported data from Spain [34] and China [35], which identified *bla*_CTX-M-14_ as the main ESBL in *E. coli* of adults with acquired UTIs. It may be noted that no acquired AmpC β-lactamase-producing *E. coli* were present in our collection, in agreement with a Portuguese study [36], performed with both clinical and non-clinical isolates [37,38,39].

The global spread of *E. coli* isolates producing *bla*_CTX-M-27_ was recently reported [40]_._ While these have been found in different parts of the world such as Germany, Spain, France, Japan, and China, they were not identified among the isolates analyzed in this study [10,35,41,42,43].

Findings of our study showed that two patients with UTIs caused by ESBL-MDR-EC recurred with ESBL-profile in the subsequent episode, in agreement with Ahn et al. [44] who found that out of 60 patients, 43 (71.7%) recurred with ESBL-producing *E. coli* in the first UTI recurrence episode.

The other ESBL enzyme found was SHV-12, which has been described worldwide as one of the most common SHV-type ESBL found in *Enterobacteriaceae* [1]. In the light of these results, we conclude that ESBLs, CTX-M-14, CTX-M-15, and SHV-12 were predominant in the Hospital de Cerdanya, similarly to what was previously described in a tertiary-care hospital in Barcelona [45].

CipR was noticed in most of the ESBL-MDR-EC. This is in agreement with other reports from India, Saudi Arabia, and Spain, which found a strong association between ESBL-producing *Enterobacteriaceae* from UTIs and CipR [46,47,48]. Phenotypic CipR was mainly associated with known chromosomal mutations in the *gyrA* (S83L, D87N) and *parC* (S80I) genes [47,49]. In addition to these mutations, we also evidenced the quinolone resistance determinant *aac(6’)-Ib-cr*, which is increasingly found in ESBL-producing *Enterobacteriaceae* [49]. Among quinolones, ciprofloxacin is one of the most prescribed antibiotics, which might explain the linkage between CipR and carriers of *aac(6’]-Ib-cr* and CTX-M ESBL [32].

Plasmid analysis revealed that the majority of the ESBL-MDR-EC replicons were from the large size F incompatibility group (IncF), mainly in the CTX-M-14 and CTX-M-15 ESBLs-producers. Furthermore, IncF has been previously associated with the increasing emergence and global spread of *bla*_CTX-M-15_ and *aac(6’]-Ib-cr* [49,50].

Retrieved from the WGSA, we detected that most of the isolates contained more than one replicon in the CTX-M-producers; in addition to the IncF type, a variation of the IncI1 and IncX1 was evidenced. The presence of a great diversity of replicons was also described by Yasir et al. [47], who found IncF type replicons, IncY, and Col156.

Concerning VFs, all the ESBL-MDR-EC studied contained one or more VFs, which underlines the ability of *E. coli* to colonize and cause infections. Different bacterial adhesins, toxins, and iron uptakes were found. With regard to adhesins, *fimH*, *afa* (ACD), *pap*, and *iha* were detected. Extraintestinal pathogenic *E. coli* (ExPEC) are complex from a phylogenetic point of view, displaying a high genetic diversity. Nevertheless, a wide range of virulence factors (VF) have been described, and it should be noted that genome plasticity is considerable. Strains causing uncomplicated UTIs normally express several VFs, as we found in our work [51].

Additionally, we observed a high diversity of *E. coli* isolates circulating at the Cerdanya Hospital according to MLST and phylogenetic analysis, as well as the presence of the high-risk clones ST131, ST405, and ST10 [7,52]. Similar to previous reports from Tanzania [32] and Spain [53], the most common clonal complexes were ST10 and ST131, both carriers of CTX-M-14 and CTX-M-15 as the main ESBLs.

As a cross-border hospital, Hospital de Cerdanya treats both Spanish and French patients. Cerdanya is located in what has traditionally been a cattle-raising region, and recently, Day et al. reported that although these high-risk clones occur mainly in humans, ST10 has been frequently identified in isolates from cattle and poultry [9].

The epidemic clone ST131 was the most prevalent among our isolates, which included those associated with CTX-M-15 β-lactamase, fluoroquinolone resistance, and the *fimH30* allele. These features, as well as an extensive virulence gene content and ARGs, were previously found in other isolates from France and Spain [7,34]. Furthermore, *E. coli* isolates from clone ST131 belonged to the phylogenetic group B2. Other studies have shown that *E. coli* ST131/CTX-M-15/B2/*fimH30* is the most disseminated *E. coli* clonal group worldwide and that it contains an extensive antimicrobial resistance profile [36,54,55].

Isolates in this work also included CTX-M-14 β-lactamase among *E. coli* ST131, as previously described in isolates involved in community-acquired UTIs in France [41]. In addition, clones ST405, ST10, and ST95 were associated with the spread of CTX-M-3, CTX-M-15, and CTX-M-14. Previous studies also showed that ST405 contributes to the efficacious dissemination of CTX-M-15 [56,57,58], CTX-M-14 [59], and CTX-M-3 [60].

## 5. Conclusions

ESBL-MDR-EC were isolated from UTI patients diagnosed at Hospital de Cerdanya, a cross-border hospital serving a large geographical area and several municipalities. Among these isolates, there was no single predominant clone. Instead, the high-risk international clones ST131, ST405, and ST10 ESBL-MDR-EC were detected. The isolates of patients from Hospital de Cerdanya included *E. coli* ST131/CTX-M-15/B2/*fim*H30, one of the most widely disseminated *E. coli* clonal groups worldwide, which is associated with an extensive antimicrobial resistance. The most prevalent enzymes were CTX-M and TEM. Overall, our study provides further evidence of the potential for the widespread dissemination of ESBL-MDR-EC and their acquisition of ARGs circulating in the Cerdanya area.

Study limitations: Our study was based on a relatively small number of isolates and it was limited to ESBL-MDR-EC isolates of UTI. Thus, much remains to be learned about the clonal diversity of UTI-causing *E. coli* isolates, including those circulating in the area served by the Hospital de Cerdanya.

## Figures and Tables

**Table 1:** Description of the predominant sequence types (ST) and clonal complexes (CC).

Isolate	ST	CC	Serotype	Phylogroup	ESBL Genes	Other β-Lactamase Genes	Other ARG	QRDR Mutations			Plasmid Replicons	VFs
								ParC	GyrA	ParE		
6184	3944	10	O101:H9	A	*bla*_CTX-M-14_		*aac(3)-IIa*, *sul3*, *dfrA14*	S80I, A56T	S83L, D87N	S458A	IncFI, IncFII, IncI1 I	*fimH54, papC, gad, terC, cvaC,iroN, iss*, *ompT, sitA, cma, astA*
8248	1284	10	O101:H21	A	*bla*_CTX-M-15_	*bla_OXA-1,_* *bla_TEM-1B_*	*aac(3)-IIa*, *aadA5* *sul1*, *dfrA17*, *aac(6′)-Ib-cr*	S80I	S83L, D87N	S458A	IncFIA, IncFIB, IncFII	*gad, terC,iroN, iss*, *iucC, iutA*, *sitA, traT*, *astA*
8669	10	10	O101:H10	A	*bla*_CTX-M-14_			S80I, E84V	S83L, D87N	S458A	IncX1	*fimH54, cea, gad, terC*
8686	10	10	O9:H9	A	*bla*_CTX-M-182_	*bla*_TEM-1B_	*aadA5* *sul2*, *dfrA17*	S80I	S83L, D87N	S458A	IncFIB, IncFIC(FII)	*fimH54, gad, terC, cvaC, etsC, fyua, hlyF,iroN, irp2*, *iss*, *iucC, iutA, mchF, ompT, sitA, traT*
13,239	44	10	O101:H4	A	*bla*_CTX-M-15_	*bla_OXA-1,_* *bla_TEM-1B_*	*aac(3)-IIa*, *aadA2b, aadA5, sul1*, *sul3*, *dfrA17*, *aac(6′)-Ib-cr*	E84K	S83L, D87N	S458T	IncFII, IncX1	*fimH54, cea, gad, terC, iucC, iutA, sitA, traT*
13,478 ^(a)^	44	10	O101:H4	A	*bla*_CTX-M-15_	*bla_OXA-1,_* *bla_TEM-1B_*	*aac(3)-IIa*, *aadA2b, aadA5, sul1*, *sul3*, *dfrA17*, *aac(6′)-Ib-cr*	E84K	S83L, D87N	S458T	IncFII, IncX1	*fimH54, cea, gad, terC, iucC, iutA, sitA, traT*
13,816 ^(a)^	44	10	O101:H4	A	*bla*_CTX-M-15_	*bla_OXA-1,_* *bla_TEM-1B_*	*aac(3)-IIa*, *aadA2b, aadA5, sul1*, *sul3*, *dfrA17*, *aac(6′)-Ib-cr*				IncFII, IncX1	*fimH54, cea, gad, terC, iucC, iutA, sitA, traT*
7987	131	131	O25:O4	B2	*bla*_CTX-M-15_	*bla_OXA-1,_* *bla_TEM-1B_*	*aac(6′)-Ib-cr*	S80I, E84V	S83L, D87N	I529L	IncFIA, IncFIB, IncFII	*fimH30, iha, yfcV, papC,* *gad, terC, fyuA,* *irp2, iss, iucC, iutA, ompT, sitA, traT, chuA, kpsE, kpsMII_K5, usp, papA_F43, sat, hra, cnf1*
13,012	131	131	O25:O4	B2	*bla*_CTX-M-15_	*bla_OXA-1_*	*aac(3)-IIa, aadA5, sul1*, *dfrA17*, *aac(6′)-Ib-cr*	S80I, E84V	S83L, D87N	I529L	IncFIA, IncFIB, IncFII, Col156	*fimH30, iha, yfcV, papC, cea, gad, terC,* *irp2, iss, iucC, iutA, ompT, sitA, traT, chuA, kpsE, kpsMII_K5, usp, papA_F43, senB, sat, hra, cnf1*
14,307	131	131	O25:O4	B2	*bla*_CTX-M-14_	*bla_TEM-1C_*			S83L	S458A, I529L	IncFIB, IncFII, IncI1 I	*fimH161, yfcV, papC, cea, gad, terC, cvaC, etsC, fyuA, hlyF, iroN,* *irp2, iss, iucC, iutA, ompT, sitA, traT, chuA, kpsE, kpsMII_K5, usp, papA_F1, papA_F14, hra, cnf1, ibeA, mchF, cia*
6935	88	23	O8:H7	B1	*bla-_SHV-12_*		*aadA1, ant(2”)-Ia, sul1, sul2, dfrA36*	S80I	S83L, D87N	S458A	IncFIB, Inc FIC(FII), Col440II	*fimH39, lpfA, papC, afaD, afaA, afaB, afaC, afaE8, gad, terC, cvaC, etsC, fyuA, hlyF, iroN, irp2, iss, iucC, iutA, mchF, ompT, sitA, traT, hra, mcmA*
7655	88	23	O9:H17	B1	*bla-_SHV-12_*		*aadA1, ant(2”)-Ia, sul1, sul2, dfrA36*	S80I, E62K	S83L, D87N	S458A	IncFIB, Inc FIC(FII), IncI1 I	*fimH39, lpfA, papC, afaD, afaA, afaB, afaC, afaE8, gad, terC, cvaC, etsC, fyuA, hlyF, iroN, irp2, iss, iucC, iutA, mchF, ompT, sitA, traT, papA_F11 (1), hra, mcmA*
12,089 ^(b)^	405	405	O102:H6	Unknown	*bla*_CTX-M-3_	*bla_TEM-1B_*	*aph(3”)-Ib, aadA5, aph(6)-Id* *sul1, sul2, dfrA17*	S80I	S83L, D87N		IncFIA, IncFIB, IncFII	*fimH27, eilA, air, afaD, afaA, afaB, afaC, afaE,* *gad, terC, fyuA, irp2, traT, chuA, kpsE, kpsMII*
8255 ^(b)^	405	405	O102:H6	Unknown	*bla*_CTX-M-3_	*bla_TEM-1B_*	*aph(3”)-Ib, aadA5, aph(6)-Id* *sul1, sul2, dfrA17*	S80I	S83L, D87N		IncFIA, IncFIB, IncFII	*fimH27, eilA, air, afaD, afaA, afaB, afaC, afaE,* *gad, terC, fyuA, irp2, traT, chuA, kpsE, kpsMII*

^(a,b)^ Isolates from the same patient.

## Data Availability

The data used to support the findings of this study are available from the corresponding authors upon request.

## Supplementary Materials

The following supporting information can be downloaded at: https://www.mdpi.com/article/10.3390/microorganisms10030488/s1, Table S1: Description of the isolates of this study.

## References

[1] Castanheira M., Simner P.J., Bradford P.A. (2021). Extended-spectrum β-lactamases: An update on their characteristics, epidemiology and detection. JAC-Antimicrob. Resist..

[2] Paul R. (2018). State of the globe: Rising antimicrobial resistance of pathogens in urinary tract infection. J. Glob. Infect. Dis..

[3] Pitout J.D., Laupland K.B. (2008). Extended-spectrum β-lactamase-producing Enterobacteriaceae: An emerging public-health concern. Lancet Infect. Dis..

[4] Livermore D.M., Canton R., Gniadkowski M., Nordmann P., Rossolini G.M., Arlet G., Ayala J., Coque T.M., Kern-Zdanowicz I., Luzzaro F. (2006). CTX-M: Changing the face of ESBLs in Europe. J. Antimicrob. Chemother..

[5] Mazzariol A., Bazaj A., Cornaglia G. (2017). Multi-drug-resistant Gram-negative bacteria causing urinary tract infections: A review. J. Chemother..

[6] Esteve-Palau E., Solande G., Sánchez F., Sorlí L., Montero M., Güerri R., Villar J., Grau S., Horcajada J. (2015). Clinical and economic impact of urinary tract infections caused by ESBL-producing *Escherichia coli* requiring hospitalization: A matched cohort study. J. Infect..

[7] Arana D.M., Sánchez A., Bautista V., Oteo-Iglesias J., Alós J.I. (2019). ESBL-producing-multidrug resistant *E. coli* population from urinary tract infections is less diverse than non-ESBL-multidrug resistant population. Enferm. Infecc. Microbiol. Clin..

[8] Zowawi H.M., Harris P.N., Roberts M.J., Tambyah P.A., Schembri M.A., Pezzani M.D., Williamson D.A., Paterson D.L. (2015). The emerging threat of multidrug-resistant Gram-negative bacteria in urology. Nat. Rev. Urol..

[9] Day M.J., Schink A.-K., Chattaway M.A., Donascimento V., Threlfall J., Rodríguez I., Van Essen-Zandbergen A., Dierikx C., Kadlec K., Wu G. (2016). Diversity of STs, plasmids and ESBL genes among *Escherichia coli* from humans, animals and food in Germany, the Netherlands and the UK. J. Antimicrob. Chemother..

[10] Flament-Simon S.-C., Nicolas-Chanoine M.-H., García V., Duprilot M., Mayer N., Alonso M.P., García-Meniño I., Blanco J.E., Blanco M., Blanco J. (2020). Clonal Structure, Virulence Factor-encoding Genes and Antibiotic Resistance of *Escherichia coli*, Causing Urinary Tract Infections and Other Extraintestinal Infections in Humans in Spain and France during 2016. Antibiotics.

[11] Shah C., Baral R., Bartaula B., Shrestha L.B. (2019). Virulence factors of uropathogenic *Escherichia coli* (UPEC) and correlation with antimicrobial resistance. BMC Microbiol..

[12] Arana D.M., Rubio M., Alós J.I. (2017). Evolución de la multirresistencia a los antibióticos en *Escherichia coli* y *Klebsiella pneumoniae* aislados de infecciones del tracto urinario. Un análisis de 12 años (2003–2014). Enferm. Infecc. Microbiol. Clin..

[13] Cantón R., Loza E., Aznar J., Castillo F.J., Cercenado E., Fraile-Ribot P.A., González-Romo F., López-Hontangas J.L., Rodríguez-Lozano J., Suárez-Barrenechea A.I. (2019). Monitoring the antimicrobial susceptibility of Gram-negative organisms involved in intraabdominal and urinary tract infections recovered during the SMART study (Spain, 2016 and 2017). Rev. Esp. Quimioter. Publ. Of. Soc. Esp. Quimioter..

[14] Esteve-Palau E., Grau S., Herrera S., Sorlí L., Montero M., Segura C., Durán X., Horcajada J.P. (2018). Impact of an antimicrobial stewardship program on urinary tract infections caused by extended-spectrum β-lactamase-producing *Escherichia coli*. Rev. Esp. Quimioter..

[15] Sanglas A., Albarral V., Farfán M., Lorén J.G., Fusté M.C. (2017). Evolutionary Roots and Diversification of the Genus Aeromonas. Front. Microbiol..

[16] EUCAST: Clinical Breakpoints and Dosing of Antibiotics. https://eucast.org/clinical_breakpoints/.

[17] Magiorakos A.-P., Srinivasan A., Carey R.B., Carmeli Y., Falagas M.E., Giske C.G., Harbarth S., Hindler J.F., Kahlmeter G., Olsson-Liljequist B. (2012). Multidrug-resistant, extensively drug-resistant and pandrug-resistant bacteria: An international expert proposal for interim standard definitions for acquired resistance. Clin. Microbiol. Infect..

[18] Kaur J., Chopra S., Sheevani Mahajan G. (2013). Modified double disc synergy test to detect ESBL production in urinary isolates of *Escherichia coli* and *Klebsiella pneumoniae*. J. Clin. Diagn. Res..

[19] Bankevich A., Nurk S., Antipov D., Gurevich A.A., Dvorkin M., Kulikov A.S., Lesin V.M., Nikolenko S.I., Pham S., Prjibelski A.D. (2012). SPAdes: A new genome assembly algorithm and its applications to single-cell sequencing. J. Comput. Biol..

[20] Parks D.H., Imelfort M., Skennerton C.T., Hugenholtz P., Tyson G.W. (2015). CheckM: Assessing the quality of microbial genomes recovered from isolates, single cells, and metagenomes. Genome Res..

[21] Bortolaia V., Kaas R.S., Ruppe E., Roberts M.C., Schwarz S., Cattoir V., Philippon A., Allesoe R.L., Rebelo A.R., Florensa A.F. (2020). ResFinder 4.0 for predictions of phenotypes from genotypes. J. Antimicrob. Chemother..

[22] Zankari E., Allesøe R., Joensen K.G., Cavaco L.M., Lund O., Aarestrup F.M. (2017). PointFinder: A novel web tool for WGS-based detection of antimicrobial resistance associated with chromosomal point mutations in bacterial pathogens. J. Antimicrob. Chemother..

[23] Camacho C., Coulouris G., Avagyan V., Ma N., Papadopoulos J., Bealer K., Madden T.L. (2009). BLAST+: Architecture and applications. BMC Bioinform..

[24] Carattoli A., Hasman H. (2019). PlasmidFinder and In Silico pMLST: Identification and Typing of Plasmid Replicons in Whole-Genome Sequencing (WGS). Horizontal Gene Transfer.

[25] Joensen K.G., Tetzschner A.M., Iguchi A., Aarestrup F.M., Scheutz F. (2015). Rapid and Easy In Silico Serotyping of *Escherichia coli* Isolates by Use of Whole-Genome Sequencing Data. J. Clin. Microbiol..

[26] Spurbeck R.R., Dinh P.C., Walk S.T., Stapleton A.E., Hooton T.M., Nolan L.K., Kim K.S., Johnson J.R., Mobley H.L.T. (2012). *Escherichia coli* Isolates That Carry vat, fyuA, chuA, and yfcV Efficiently Colonize the Urinary Tract. Infect. Immun..

[27] Roer L., Tchesnokova V., Allesøe R., Muradova M., Chattopadhyay S., Ahrenfeldt J., Thomsen M.C.F., Lund O., Hansen F., Hammerum A.M. (2017). Development of a Web Tool for *Escherichia coli* Subtyping Based on fimH Alleles. J. Clin. Microbiol..

[28] Zhou Z., Alikhan N.-F., Mohamed K., Fan Y., Achtman M., Brown D., Chattaway M., Dallman T., Delahay R., the Agama Study Group (2020). The EnteroBase user’s guide, with case studies on Salmonella transmissions, Yersinia pestis phylogeny, and Escherichia core genomic diversity. Genome Res..

[29] Treangen T.J., Ondov B.D., Koren S., Phillippy A.M. (2014). The Harvest suite for rapid core-genome alignment and visualization of thousands of intraspecific microbial genomes. Genome Biol..

[30] Letunic I., Bork P. (2021). Interactive Tree Of Life (iTOL) v5: An online tool for phylogenetic tree display and annotation. Nucleic Acids Res..

[31] Rodríguez-Baño J., Alcalá J.C., Cisneros J.M., Grill F., Oliver A., Horcajada J.P., Tórtola T., Mirelis B., Navarro G., Cuenca M. (2008). Community Infections Caused by Extended-Spectrum β-Lactamase–Producing *Escherichia coli*. Arch. Intern. Med..

[32] Sonda T., Kumburu H., van Zwetselaar M., Alifrangis M., Mmbaga B.T., Aarestrup F.M., Kibiki G., Lund O. (2018). Whole genome sequencing reveals high clonal diversity of *Escherichia coli* isolated from patients in a tertiary care hospital in Moshi, Tanzania. Antimicrob. Resist. Infect. Control.

[33] Oteo J., Campos J., Baquero F. (2002). Antibiotic resistance in 1962 invasive isolates of *Escherichia coli* in 27 Spanish hospitals participating in the European Antimicrobial Resistance Surveillance System (2001). J. Antimicrob. Chemother..

[34] Hernández-García M., García-Fernández S., García-Castillo M., Bou G., Cercenado E., Delgado-Valverde M., Mulet X., Pitart C., Rodríguez-Lozano J., Tormo N. (2020). WGS characterization of MDR Enterobacterales with different ceftolozane/tazobactam susceptibility profiles during the SUPERIOR surveillance study in Spain. JAC-Antimicrob. Resist..

[35] Jia P., Zhu Y., Li X., Kudinha T., Yang Y., Zhang G., Zhang J., Xu Y., Yang Q. (2021). High Prevalence of Extended-Spectrum Beta-Lactamases in *Escherichia coli* Strains Collected From Strictly Defined Community-Acquired Urinary Tract Infections in Adults in China: A Multicenter Prospective Clinical Microbiological and Molecular Study. Front. Microbiol..

[36] Carvalho I., Carvalho J.A., Martínez-Álvarez S., Sadi M., Capita R., Alonso-Calleja C., Rabbi F., Dapkevicius M.D.L.N.E., Igrejas G., Torres C. (2021). Characterization of ESBL-Producing *Escherichia coli* and *Klebsiella pneumoniae* Isolated from Clinical Samples in a Northern Portuguese Hospital: Predominance of CTX-M-15 and High Genetic Diversity. Microorganisms.

[37] Ribeiro T.G., Novais Â., Machado E., Peixe L. (2020). Acquired AmpC β-Lactamases among Enterobacteriaceae from Healthy Humans and Animals, Food, Aquatic and Trout Aquaculture Environments in Portugal. Pathogens.

[38] Ribeiro T., Novais Â., Rodrigues C., Nascimento R., Freitas F., Machado E., Peixe L. (2019). Dynamics of clonal and plasmid backgrounds of Enterobacteriaceae producing acquired AmpC in Portuguese clinical settings over time. Int. J. Antimicrob. Agents.

[39] Rodríguez-Navarro J., Miró E., Brown-Jaque M., Hurtado J.C., Moreno A., Muniesa M., González-López J.J., Vila J., Espinal P., Navarro F. (2020). Comparison of Commensal and Clinical Isolates for Diversity of Plasmids in *Escherichia coli* and *Klebsiella pneumoniae*. Antimicrob. Agents Chemother..

[40] Castanheira M., Doyle T.B., Mendes R.E., Sader H.S. (2019). Comparative activities of ceftazidime-avibactam and ceftolozanetazobactam against *Enterobacteriaceae* isolates producing extended-spectrum β-lactamases from U.S. hospitals. Antimicrob. Agents Chemother..

[41] Birgy A., Madhi F., Jung C., Levy C., Cointe A., Bidet P., Hobson C.A., Bechet S., Sobral E., Vuthien H. (2019). Diversity and trends in population structure of ESBL-producing Enterobacteriaceae in febrile urinary tract infections in children in France from 2014 to 2017. J. Antimicrob. Chemother..

[42] Matsumura Y., Johnson J.R., Yamamoto M., Nagao M., Tanaka M., Takakura S., Ichiyama S., Fujita N., Komori T., Kyoto-Shiga Clinical Microbiology Study Group (2015). CTX-M-27- and CTX-M-14-producing, ciprofloxacin-resistant *Escherichia coli* of the H30 subclonal group within ST131 drive a Japanese regional ESBL epidemic. J. Antimicrob. Chemother..

[43] Colmenarejo C., Hernández-García M., Muñoz-Rodríguez J.R., Huertas N., Navarro F.J., Mateo A.B., Pellejero E.M., Illescas S., Vidal M.D., Del Campo R. (2020). Prevalence and risks factors associated with ESBL-producing faecal carriage in a single long-term-care facility in Spain: Emergence of CTX-M-24- and CTX-M-27-producing *Escherichia coli* ST131-H30R. J. Antimicrob. Chemother..

[44] Ahn S.T., Kim S.W., Kim J.W., Park H.S., Moon D.G., Oh M.M. (2019). Does urinary tract infection caused by extended-spectrum β-lactamase-producing *Escherichia coli* show same antibiotic resistance when it recurs?. J. Infect. Chemother..

[45] Coelho A., Mora A., Mamani R., López C., González-López J.J., Larrosa M.N., Quintero-Zarate J.N., Dahbi G., Herrera A., Blanco J.E. (2010). Spread of *Escherichia coli* O25b:H4-B2-ST131 producing CTX-M-15 and SHV-12 with high virulence gene content in Barcelona (Spain). J. Antimicrob. Chemother..

[46] Usman S., Fatima S., Muhammad I.N., Jamil S., Khan M.N., Khan S.I. (2018). Incidence of multidrug resistance and extended-spectrum beta-lactamase expression in community-acquired urinary tract infection among different age groups of patients. Indian J. Pharmacol..

[47] Yasir M., Farman M., Shah M.W., Jiman-Fatani A.A., Othman N.A., Almasaudi S.B., Alawi M., Shakil S., Al-Abdullah N., Ismaeel N.A. (2020). Genomic and antimicrobial resistance genes diversity in multidrug-resistant CTX-M-positive isolates of *Escherichia coli* at a health care facility in Jeddah. J. Infect. Public Health.

[48] Oteo J., Lázaro E., De Abajo F.J., Baquero F., Campos J., Earss S.M.O. (2005). Antimicrobial-resistant Invasive *Escherichia coli*, Spain. Emerg. Infect. Dis..

[49] Harris P.N.A., Ben Zakour N.L., Roberts L.W., Wailan A.M., Zowawi H.M., A Tambyah P., Lye D., Jureen R., Lee T.H., Yin M. (2018). Whole genome analysis of cephalosporin-resistant *Escherichia coli* from bloodstream infections in Australia, New Zealand and Singapore: High prevalence of CMY-2 producers and ST131 carrying blaCTX-M-15 and blaCTX-M-27. J. Antimicrob. Chemother..

[50] Marcadé G., Deschamps C., Boyd A., Gautier V., Picard B., Branger C., Denamur E., Arlet G. (2009). Replicon typing of plasmids in *Escherichia coli* producing extended-spectrum β-lactamases. J. Antimicrob. Chemother..

[51] Sarowska J., Futoma-Koloch B., Jama-Kmiecik A., Frej-Madrzak M., Ksiazczyk M., Bugla-Ploskonska G., Choroszy-Krol I. (2019). Virulence factors, prevalence and potential transmission of extraintestinal pathogenic *Escherichia coli* isolated from different sources: Recent reports. Gut Pathog..

[52] Merida-Vieyra J., De Colsa-Ranero A., Calderón-Castañeda Y., Aquino-Andrade A. (2020). Detection of CMY-type beta-lactamases in *Escherichia coli* isolates from paediatric patients in a tertiary care hospital in Mexico. Antimicrob. Resist. Infect. Control.

[53] Oteo J., Diestra K., Juan C., Bautista V., Novais Â., Perez-Vazquez M., Moyá B., Miro E., Coque T.M., Oliver A. (2009). Extended-spectrum β-lactamase-producing *Escherichia coli* in Spain belong to a large variety of multilocus sequence typing types, including ST10 complex/A, ST23 complex/A and ST131/B2. Int. J. Antimicrob. Agents.

[54] Belas A., Marques C., Aboim C., Pomba C. (2018). Emergence of *Escherichia coli* ST131 H30/H30-Rx subclones in companion animals. J. Antimicrob. Chemother..

[55] Zhang L., Foxman B., Marrs C. (2002). Both Urinary and Rectal *Escherichia coli* Isolates Are Dominated by Strains of Phylogenetic Group B_2_. J. Clin. Microbiol..

[56] Ruiz S.J., Montealegre M.C., Ruiz-Garbajosa P., Correa A., Briceño D.F., Martinez E., Rosso F., Muñoz M., Quinn J.P., Cantón R. (2011). First Characterization of CTX-M-15-Producing *Escherichia coli* ST131 and ST405 Clones Causing Community-Onset Infections in South America. J. Clin. Microbiol..

[57] Coque T.M., Novais Â., Carattoli A., Poirel L., Pitout J., Peixe L., Baquero F., Cantón R., Nordmann P. (2008). Dissemination of Clonally Related *Escherichia coli* Strains Expressing Extended-Spectrum β-Lactamase CTX-M-15. Emerg. Infect. Dis..

[58] Rogers B.A., Sidjabat H.E., Paterson D.L. (2010). *Escherichia coli* O25b-ST131: A pandemic, multiresistant, community-associated strain. J. Antimicrob. Chemother..

[59] Shin J., Kim D.H., Ko K.S. (2011). Comparison of CTX-M-14- and CTX-M-15-producing *Escherichia coli* and *Klebsiella pneumoniae* isolates from patients with bacteremia. J. Infect..

[60] Mihaila L., Wyplosz B., Clermont O., Garry L., Hipeaux M.C., Vittecoq D., Dussaix E., Denamur E., Branger C. (2010). Probable intrafamily transmission of a highly virulent CTX-M-3-producing *Escherichia coli* belonging to the emerging phylogenetic subgroup D2 O102-ST405 clone. J. Antimicrob. Chemother..

